# Machine learning-based prediction model using clinico-pathologic factors for papillary thyroid carcinoma recurrence

**DOI:** 10.1038/s41598-021-84504-2

**Published:** 2021-03-02

**Authors:** Young Min Park, Byung-Joo Lee

**Affiliations:** 1 Department of Otorhinolaryngology, Gangnam Severance Hospital, Yonsei University College of Medicine, 211 Eonju-ro, Gangnam-gu, Seoul, 06273 Korea; 2 Department of Otorhinolaryngology-Head and Neck Surgery, Pusan National University School of Medicine, Pusan National University and Biomedical Research Institute, Pusan National University Hospital, 1-10 Ami-Dong, Seo-Gu, Busan, 602-739 Korea

**Keywords:** Medical research, Oncology, Risk factors

## Abstract

This study analyzed the prognostic significance of clinico-pathologic factors, including the number of metastatic lymph nodes (LNs) and lymph node ratio (LNR), in patients with papillary thyroid carcinoma (PTC), and attempted to construct a disease recurrence prediction model using machine learning techniques. We retrospectively analyzed clinico-pathologic data from 1040 patients diagnosed with PTC between 2003 and 2009. We analyzed clinico-pathologic factors related to recurrence through logistic regression analysis. Among the factors that we included, only sex and tumor size were significantly correlated with disease recurrence. Parameters such as age, sex, tumor size, tumor multiplicity, ETE, ENE, pT, pN, ipsilateral central LN metastasis, contralateral central LNs metastasis, number of metastatic LNs, and LNR were input for construction of a machine learning prediction model. The performance of five machine learning models related to recurrence prediction was compared based on accuracy. The Decision Tree model showed the best accuracy at 95%, and the lightGBM and stacking model together showed 93% accuracy. Among those factors mentioned above, LNR and contralateral LN metastasis were used as important features in all machine learning prediction models. We confirmed that all machine learning prediction models showed an accuracy of 90% or more for predicting disease recurrence in PTC. LNR and contralateral LN metastasis were used as important features for constructing a robust machine learning prediction model. In the future, we have a plan to perform large-scale multicenter clinical studies to improve the performance of our prediction models and verify their clinical effectiveness.

## Introduction

Over the past 20 years, the incidence of thyroid cancer has increased rapidly, and the most common histologic type of thyroid cancer is papillary thyroid carcinoma (PTC)^1^. PTC has an excellent prognosis and a better survival rate than other carcinomas;however, disease ultimately recurs in about 5–21% of PTC patients^2,3^. In PTC patients with recurrent disease, surgical treatment is generally required, and re-operation poses additional medical costs and significant morbidity, compared to the initial surgery. Therefore, preventing recurrence in PTC patients can reduce and deteriorations in quality of life related to reoperation.

According to previous reports, tumor size, extrathyroidal extension (ETE), age, lymph node (LN) metastasis, tumor multiplicity, and extranodal spread (ENE) are known risk factors for recurrence of PTC^4–6^. In PTC, LN metastasis occurs in 20–90% of patients, and the number of metastatic LNs and lymph node ratio (LNR), representing metastatic LN burden, are known to be important prognostic factors associated with recurrence of PTC^7–13^. Since various clinico-pathological factors and nodal factors (i.e., number of metastatic LNs and LNR) have been shown to be related to the recurrence of PTC, these factors should be considered in an integrated manner to establish a disease recurrence prediction model.

The 8th TNM staging system was revised by the American Joint Committee on Cancer (AJCC) to more accurately predict the disease-specific survival of PTC patients. However, it does not reflect the biological behavior of PTC and has limitations in predicting the risk of recurrence^14–16^. In particular, the number and size of metastatic LNs are not reflected in the revised TNM staging system. Also, the N classification of the revised TNM stage system merely comprises three groups and does not consider other nodal factors^17–22^. We presume that the more prognostic factors, such as number of metastatic LNs and LNR, that can be integrated into the TNM system, the more accurate the prediction model could be for predicting disease recurrence of PTC patients.

Machine learning technology is widely used in the medical field, especially in the fields of radiology, ophthalmology, and dermatology^23–28^. However, studies on the construction of machine learning models that can be used to predict disease recurrence of PTC are extremely rare. Establishing a robust predictive model of PTC could help with selecting high-risk patients for intensified treatment tailored according to risk stratification and with suggesting candidates for active follow-up. This study analyzed the prognostic significance of various clinico-pathologic nodal factors, including the number of metastatic LNs and LNR, in patients with PTC patients and attempted to construct a disease recurrence prediction model based on these factors using machine learning techniques.

## Materials and methods

This study was approved by the Institutional Review Board (IRB) of Pusan University. Informed consent was not obtained from any participants because the IRB waived the need for individual informed consent. This retrospective research was performed in accordance with the Declaration of Helsinki. Medical data of patients diagnosed and treated for PTC at Pusan National University Hospital from June 2003 to December 2009 were analyzed retrospectively. We included patients who were diagnosed with PTC and underwent total thyroidectomy and central neck dissection with/without lateral neck dissection. We excluded (1) cases with a distant metastasis at the time of diagnosis, (2) patients who received previous surgery or radiotherapy to the head and neck area, and (3) cases with insufficient clinical data that were lost to follow-up after surgery. Finally, 1040 patients were included in the study, including 147 males and 893 females. Their ages ranged from 13 to 79 years and the mean age was 48.5 years. Tumor stage was classified according to the 8th AJCC staging system.

To detect disease recurrence, all patients underwent physical examination, ultrasound, and thyroglobulin measurement every 6–12 months after surgery. If necessary, additional imaging studies such as computed tomography, whole body iodine scan, and positron emission tomography were performed. Recurrence was defined as a case in which a new lesion was detected that was not previously observed in the imaging studies and which was pathologically confirmed through fine needle aspiration cytology.

Tumor size, ETE, multiplicity, ENE, and TNM stage were analyzed. The surgical specimens from central neck dissection were divided into ipsilateral and contralateral areas according to the location of the tumor, and therein the number of metastatic LNs and the total number of harvested LNs were recorded. LNR was calculated by dividing the number of metastatic LNs by the total number of harvested LNs. The cut-off value of LNR was determined in consideration of optimized sensitivity and specificity for predicting disease recurrence using receiver operating characteristic curves (ROC). The optimal value of LNR was used to construct machine learning models for predicting disease recurrences.

Machine learning was performed based on the supervised learning method, and a variety of machine learning models was used including the decision tree, random forest, XGBoost, and LightGBM, and Stacking models. Learning was performed with the five models mentioned above, and accuracy was used to evaluate model performance. Scikit-learn version 12.3 was used for model building and learning. 80% of the data set was classified as the training set and was used for learning, and the remaining 20% was used as a test set. To account for selection bias, the five-fold-cross-validation technique was applied.

Patient clinical information, pathologic information, recurrence, and cause of recurrence were analyzed. The Chi-square or independent two-sample t-test were used to evaluate differences in variables between two independent groups. The multivariate Cox proportional hazards regression model was used to evaluate the effect of several variables on disease recurrence. *p* values < 0.05 were considered indicative of statistical significance. Statistical analyses were performed using python 3.8 version and SPSS version 25.0 for Windows (SPSS, Chicago, IL).

## Results

A total of 1040 patients were included in this study, and all patients underwent total thyroidectomy and central neck dissection. In total, 180 patients (17.3%) underwent lateral neck dissection simultaneously due to lateral LN metastasis. The average tumor size was 12.4 mm (range 2–125). ETE findings were observed in 586 (56.3%) patients, and tumor multiplicity was observed in 178 (17.1%) patients. ENE findings were observed in 159 patients (15.3%). With respect to T classification, 508 patients were classified as T1, 46 patients were T2, 483 patients were T3, and three patients were T4. The average number of metastatic LNs in the central compartment was 1.73 (range 0–19), and the average number of LNs removed was 8.32 (range 0–36). The average LNR value was 0.20 (range 0–1). The mean follow-up period was 79.0 months (range 46–149), and the total number of recurrence events during the study period was 41. Other clinico-pathological information is summarized in Table 1.

**Table 1:** Clinical information for all patients (n = 1040) enrolled in the study.

Variable	No. of patients (%)
Mean age, y (range)	48.5 (13–79)
**Sex**	
Male	147 (14.1)
Female	893 (85.9)
**Extrathyroidal extension**	
Yes	586 (56.3)
No	454 (46.7)
**Tumor multiplicity**	
Yes	178 (17.1)
No	862 (82.9)
**Extranodal extension**	
Yes	159 (15.3)
No	881 (84.7)
**pT classification**	
1	508 (48.8)
2	46 (4.4)
3	483 (46.4)
4	3 (0.2)
**pN classification**	
N0	506 (48.7)
N1a	354 (34.0)
N1b	180 (17.3)

**Figure 1:**
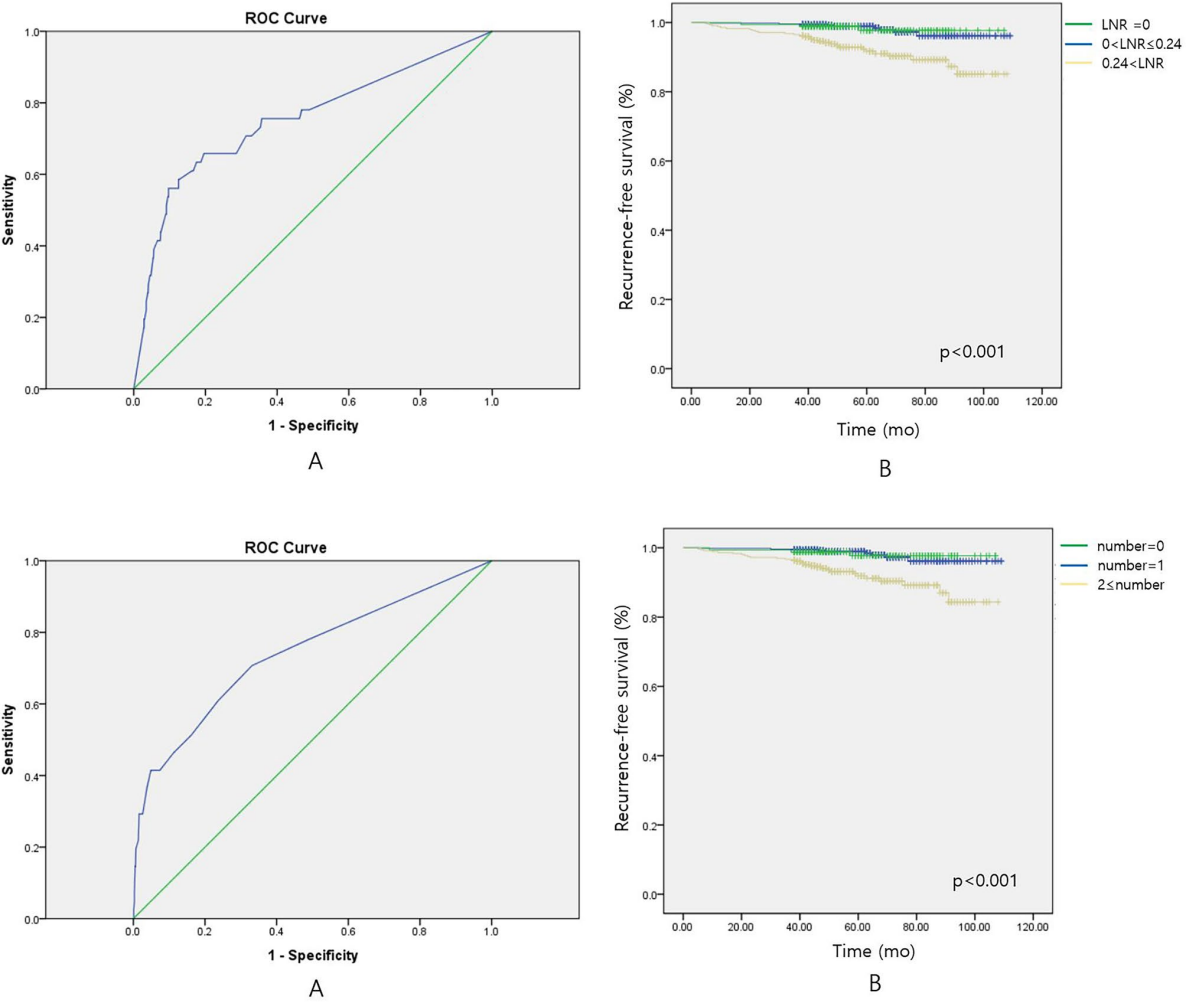
(**A**) The cut-off value for the lymph node ratio (LNR) was determined in consideration of the sensitivity and specificity optimized to predict disease recurrence using a receiver operating characteristic curve (ROC). Recurrence-free survival of the patient group with LNR > 0.24 was significantly decreased. (**B**) The cut-off value for the number of metastatic LNs was determined in consideration of the sensitivity and specificity optimized to predict disease recurrence using a ROC. The recurrence-free survival of the patient group with two or more metastatic LNs was significantly decreased. (The graph was made by statistical software SPSS version 25.0 for windows).

The cut-off value for the LNR was set to show the optimal sensitivity and specificity for recurrence prediction. Regarding the prediction of recurrence, LNR showed a statistically significant correlation (*p* value =  < 0.001), with an AUC value of 0.752, and an LNR value of 0.24 was set as the optimal cut-off value. There were 519 patients (49.9%) with an LNR-of 0; 179 (17.2%) with an LNR value greater than 0, but less than 0.24; and 342 (32.9%) with an LNR value of 0.24 or more. Recurrence-free survival was significantly lower in the patient group with LNR > 0.24, compared to the other two groups (Fig. 1A). A cut-off value for the number of metastatic LNs was devised to obtain optimal sensitivity and specificity for recurrence prediction. The number of metastatic LNs was statistically significantly correlated with recurrence (*p* value =  < 0.001), with an AUC value of 0.742. A value of 2 was set as the cut-off for the number of metastatic LNs. There were 519 patients (49.9%) with 0 metastatic LNs, 161 (15.5%) with one LN metastasis, and 360 (34.6 with two or more LN metastases. Recurrence-free survival was analyzed by dividing patients into these three groups and was significantly decreased lower in patients with two or more metastatic LNs (Fig. 1B).

We analyzed the association between clinico-pathologic factors and recurrence through univariate analysis. Sex, tumor size, ETE, pT classification, pN classification, number of metastatic LNs, and LNR were significantly correlated with disease recurrence. Clinico-pathologic factors related to recurrence were also analyzed with logistic regression for multivariate analysis. Among the factors included in the analysis, only sex and tumor size showed significant correlations with disease recurrence (Table 2).

**Table 2:** Logistic regression analysis for disease recurrence adjusted for clinico-pathologic factors.

Factor	Hazard ratio	95% CI	*p* value
Sex	2.393	1.128–5.077	0.023
Size	1.030	1.007–1.052	0.010
ETE	3.105	0.697–13.834	0.137
pT	1.547	0.810–2.954	0.186
pN	0.790	0.170–3.683	0.765
Number of metastatic CLNs	1.619	0.582–4.502	0.356
LNR	1.337	0.480–3.725	0.578

*CI* confidence interval, *ETE* extrathyroidal extension, *LN* lymph node, *LNR* lymph node ratio.

**Figure 2:**
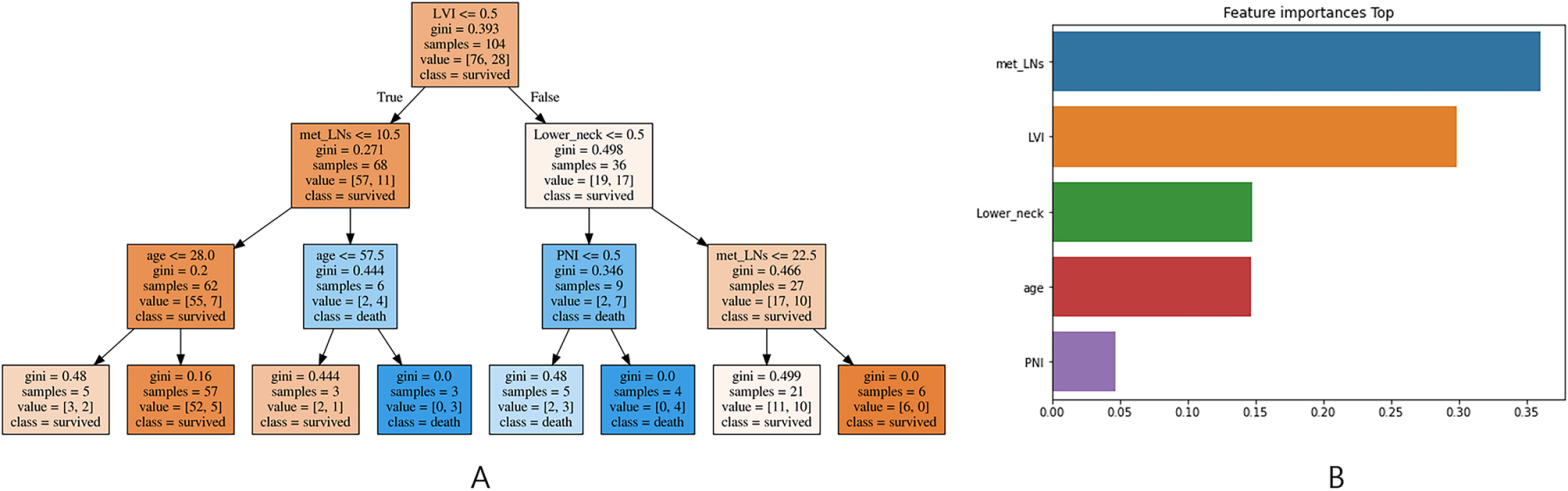
The structure of the decision tree model was visualized using graphic software. (The graph was made using by python version 3.8, scikit-learn version 12.3, and graphviz software, All these software are open-sourec program).

To build a machine learning prediction model, the algorithm was trained using parameters of age, sex, tumor size, tumor multiplicity, ETE, ENE, pT, pN, ipsilateral central LN metastasis, contralateral central LN metastasis, number of metastatic LNs, and LNR. Since disease recurred in only 41 of 1040 cases, the SMOTE technique was applied to adjust for the imbalance in learning data. The performance of five machine learning models for recurrence prediction was compared based on accuracy. The decision tree model showed the best accuracy at 95%, and the LightGBM and stacking models showed accuracies of 93%. Table 3 summarizes the performance of the five models. The tree structure of the decision tree model was visualized using graphic software, and feature importance was also visualized and analyzed (Fig. 2). In other machine learning models, feature importance was explored to determine the major factors that influence the prediction of recurrence in PTC patients. Although the feature importance results differed slightly between machine learning models, LNR and contralateral LN metastasis were consistently important features in all models (Table 4).

**Table 3:** Performance results for the machine learning models.

Model	Accuracy	Precision	Recall	F1 score
Decision tree	0.95	0.66	0.18	0.28
Random forest	0.91	0.25	0.27	0.26
XGBoost	0.92	0.25	0.18	0.21
LightGBM	0.93	0.28	0.18	0.22
Stacking	0.93	0.33	0.18	0.23

**Table 4:** Top five features of importance among machine learning models.

Rank	Decision tree	Random forest	XGBoost	LightGBM
1st	Contralateral CLN metastasis	LNR	Tumor size	Age
2nd	LNR	Ipsilateral CLNs metastasis	Age	Tumor size
3rd	Age	Contralateral CLNs metastasis	Ipsilateral CLNs metastasis	Ipsilateral CLNs metastasis
4th		Tumor size	LNR	LNR
5th		Age	Contralateral CLNs metastasis	Contralateral CLNs metastasis

*LN* lymph node, *LNR* lymph node ratio.

## Discussion

The revised 8th TNM staging system is suitable for assessing the risk of death in patients with PTC, but not for predicting the risk of recurrence. Age, aggressive histology, tumor size, and LNs metastasis are known risk factors associated with PTC recurrence^29^. The 2015 American Thyroid Association guidelines suggested the number and size of metastatic LNs and ENE as risk factors for recurrence^30,31^. Meanwhile, LNR, calculated by dividing the number of metastatic LNs by the total number of removed LNs, has been reported in previous studies as a risk factor for recurrence of PTC^13,32,33^. Lee et al.^34^ reported that the performance of recurrence prediction increased when LNR was incorporated into the existing 2015 ATA risk stratification. In our study, the sensitivity and specificity for predicting disease recurrence were optimized when an LNR value of 0.24 was set as a cut-off. The number of metastatic LNs also showed a statistically significant correlation with the prediction of disease recurrence when 2 or more LNs was set as the cut-off value. To construct a robust prediction model for disease recurrence in PTC, these all factors mentioned above should be considered and integrated into the model.

In univariate analysis of risk factors for PTC recurrence, sex, tumor size, ETE, pT, pN, number of metastatic LNs, and LNR were significantly correlated with recurrence. In multivariate analysis using logistic regression, only tumor size showed a significant correlation with disease recurrence. Because logistic regression was used to analyze prognostic factors based on a linear combination between variables, if the degree of correlation between variables was high, the analysis was limited with overfitted results. On the other hand, since machine learning models do not assume a linear combination of variables used, the effect of correlation between variables can be diminished. Therefore, we can put all those prognostic factors including nodal factors such as LNR and number of metastatic LNs into the model and constructed machine learning prediction model. When analyzing the feature importance of parameters used for machine learning model construction, we noted that contralateral LN metastasis and LNR were used at high frequencies for machine learning model construction in all machine learning models, along with other clinical factors, including tumor size and age.

Among the machine learning techniques used in this study, the decision tree model showed the highest accuracy, followed by the Ensemble models, including LightGBM and stacking techniques. All of the machine learning models showed accuracies of 90% or more. However, since the models were trained based on data from 1000 patients, more patient data would be required to increase the performance of our models and to apply them in clinical practice. Also, additional research is needed to address the following limitations: Since this was a retrospective study conducted at a single institution, the influence of selection bias cannot be excluded. In addition, considering the indolent features of PTC, a short follow-up period is less optimal for detecting recurrence in PTC patients. Nevertheless, this study is of value as the first study on machine learning models of predicting PTC disease recurrence based clinico-pathologic factors.

In this study, various machine learning models were constructed for predicting disease recurrence in PTC patients, and all of the models had a confirmed accuracy of 90% or more. In the future, large-scale clinical studies on many patients should be performed to improve the performance of our prediction models, and multicenter clinical studies will be needed to verify their clinical effectiveness.
